# The Ameliorating Effect of Berberine-Rich Fraction against Gossypol-Induced Testicular Inflammation and Oxidative Stress

**DOI:** 10.1155/2018/1056173

**Published:** 2018-04-03

**Authors:** Samar R. Saleh, Rana Attia, Doaa A. Ghareeb

**Affiliations:** ^1^Biochemistry Department, Faculty of Science, Alexandria University, Alexandria, Egypt; ^2^Pharmaceutical and Fermentation Industries Development Centre, City for Scientific Research and Technology Applications, Alexandria, Egypt; ^3^Biological Sciences Department, Faculty of Science, Beirut Arab University, Beirut, Lebanon

## Abstract

This study was aimed at evaluating the efficacy of berberine-rich fraction (BF) as a protective and/or a therapeutic agent against inflammation and oxidative stress during male infertility. Sexually mature Sprague-Dawley male rats were divided into five groups treated with either corn oil, BF (100 mg/kg BW, orally, daily for 30 days), gossypol acetate (5 mg/kg BW, i.p.) eight times for 16 days, BF alone for 14 days then coadministered with gossypol acetate for the next 16 days (protected group), or gossypol acetate for 16 days then treated with BF for 30 days (treated group). All animals completed the experimental period (46 days) without obtaining any treatments in the gap period. Sperm parameters, oxidative index, and inflammatory markers were measured. Gossypol injection significantly decreased the semen quality and testosterone level that resulted from the elevation of testicular reactive oxygen and nitrogen species (TBARS and NO), TNF-*α*, TNF-*α*-converting enzyme, and interleukins (IL-1*β*, IL-6, and IL-18) by 230, 180, 12.5, 97.9, and 300%, respectively, while interleukin-12 and tissue inhibitors of metalloproteinases-3 were significantly decreased by 59 and 66%, respectively. BF (protected and treated groups) significantly improved the semen quality, oxidative stress, and inflammation associated with male infertility. It is suitable to use more advanced studies to validate these findings.

## 1. Introduction

Infertility is the disability of a couple to achieve pregnancy after one year without intercourse precautions [1]. Male infertility is found to contribute 45%–50% of infertility cases [2]. Africa and Central/Eastern Europe were considered to have the highest rates of infertility [3]. Many factors are known to impair male infertility, including varicocele, testicular failure, treatment with radiation, and illicit drugs [4]. Infertility produces psychological stress as a couple fails achieve the expected goal of reproduction, causing disappointment and frustration that is attributed to generalized increased oxidative stress levels [5]. Oxidative stress is a well-known causative factor involved in the etiology of male infertility [6, 7]. Oxidative stress arises when reactive oxygen species (ROS) or free radical production overwhelms the endogenous antioxidant defense of the male reproductive tract [1, 8]. ROS have destructive effects on semen quality by disrupting the integrity of sperm nuclear DNA and ATP production [1]. The spermatozoal cell membrane contains a high abundance of polyunsaturated fatty acids representing the most vulnerable target for free radical damage and lipid peroxidation and, hence, influencing the sperm viability, count, motility, and morphology [9]. Inflammation could be linked to oxidative stress, and as oxidative stress primarily occurs, it can further induce inflammation and vice versa; thus, they strengthen each other, causing destructive effects to the cells [10, 11].

Cytokines, including interleukins (ILs) and tumor necrosis factor alpha (TNF-*α*), are important mediators of immunity and can be contributed in numerous physiological processes in the male reproductive tract [2]. Cytokines have different effects on the semen quality and sperm function. IL-1*β*, IL-6, and IL-18 are proinflammatory cytokines, can be produced by specific cells in the male reproductive system (such as testicular somatic cells and Sertoli cells), are included in the inflammatory reaction, and can induce apoptosis [2], while IL-12 improves male fertility due to its immunomodulatory properties [12]. It is involved in the induction and maintenance of the immune response during both cell-mediated (helper T1) and humoral responses (helper T2) [13] and regulating antigen-presenting activity and natural killer cell activity [14]. TNF-*α* has destructive effects on sperm [8], and the TNF-*α*-converting enzyme (TACE; ADAM-17) is an enzyme involved in the proteolytic liberation of TNF-*α* from the pro-TNF-*α* molecule. Since ADAM-17 was found to be inhibited by tissue inhibitors of metalloproteinases-3 (TIMP-3); TIMP-3 are important factors involved in the regulation of the inflammatory process and the disease progression [15].

Antioxidants quench free radicals and protect gonadal cells and mature spermatozoa from ROS production and oxidative damage [9]. According to the World Health Organization (WHO), developing countries make use of herbal medicinal products for a variety problems due to their safety and the side effects of chemical drugs [16]. Berberine is an isoquinoline alkaloid that belongs to the structural class of protoberberines [17] and is present in roots, rhizomes, and stem bark of the *Berberis* species that belongs to the Berberidaceae family. Berberine is the most active constituent in *Berberis vulgaris* [18–20]. Several studies have indicated that berberine acts as a natural medicine with multiple biochemical and pharmacological activities [21, 22] including anti-inflammatory [23], antioxidant [24], antidepressant [25, 26], anticancer [27], hypoglycemic, hypolipidemic [22], and antimicrobial activities [19].

Gossypol was used as antifertility agent in male rats. Gossypol is a very toxic crystalline polyphenolic compound and is found in the highest concentration in the seeds of cotton plants [28]. Gossypol induces oxidative stress by the imbalance between antioxidants and prooxidants, resulting in the accumulation of ROS [29]. Oral gossypol acetate was found to reduce the levels of serum testosterone and luteinizing hormone in a dose- and duration-dependent manner [30]. Gossypol acts directly on testes and induces azoospermia or oligospermia [31]. Furthermore, gossypol blocked cAMP formation in sperms, which subsequently decreased sperm motility [32]. It also reduced the secretory activity of accessory sex glands [33]. Therefore, gossypol was used as an efficient male contraceptive drug [34].

The present study was aimed at assessing the therapeutic and/or protective effects of BF against the inflammation process produced during male infertility induced in rats by using gossypol acetate. The study will demonstrate its effect on biochemical blood parameters (TBARS, GSH, testosterone, cholesterol, glucose, and albumin), semen quality (sperm count, motility, morphology, *α*- glucosidase activity, and fructose level), and finally the inflammatory markers [testicular TBARS, NO, TNF-*α*, ADAM-17, TIMP-3, and interleukins (IL-1*β*, IL-6, IL-12, and IL-18)].

## 2. Materials and Methods

### 2.1. Plant Collection and Preparation of Ethanol Extract and Different Fractions

Barberry roots were purchased and authenticated by Professor Salma El-dareir, Botany Department, Faculty of Science, Alexandria University, Egypt. This classification was dependent on the data about the plant published in the Dargon Herbarium. Barberry roots were subjected to steam distillation, and the ethanol extract was prepared as described by [27]. The extract was lyophilized and the obtained powder (35 g, ethanolic extract) was dissolved in 1% HCl and then filtrated. The pH of the filtrate was optimized to 8 by using concentrated NH_4_OH. The tertiary alkaloids were extracted from the previous solution by using chloroform, and this fraction (chloroform fraction) was evaporated and lyophilized (25 g). The obtained powder was dissolved in the minimum amount of chloroform and then was subjected to a silica gel 100–200 mesh column. A berberine-rich fraction (2.5 g) was obtained by using gradient elution with CHCl_3_ : MeOH (9 : 1; 8 : 2) and finally methanol [35]. The presence of berberine in the fraction was identified using TLC, melting point [36], HPLC [27], and ^1^H-NMR [37]. The obtained powder was dissolved in polyethylene glycol (20%) to be administrated to rats.

### 2.2. Preparation of Gossypol Acetate

The Egyptian cottonseeds (Giza 70) were collected with the help of Professor Ali Aisa Nawar, Department of Crop Science, Faculty of Agriculture, Alexandria University, Egypt. The collected seeds were cleaned and crushed; the decorticated kernels were mashed by a meat chopper and finally extracted with peroxide-free ether by percolation. Briefly, the decorticated kernels (1 kg) were defatted by petroleum ether for at least 2 h at room temperature (RT), then filtrated by using a Bűchner funnel and dried. The dried kernels were soaked in peroxide-free ether at RT, overnight in the dark. The oil-ether extract was filtrated as mentioned above, and the extract was concentrated by using rotary evaporator (Büchi, Switzerland). Glacial acetic acid was added to the extract (1 : 1, v/v) and stirred thoroughly, and the gossypol acetate crystals were precipitated overnight. The crystals were collected and stored in refrigerator until use [38].

### 2.3. Animal Experimental Design

Thirty albino sexually mature Sprague-Dawley male rats, about 10–12 weeks of age (100–130 g body weight), were purchased from the experimental animal house of the Faculty of Science, Cairo University, and housed in the animal house of the physiology department of the Faculty of Medicine, Alexandria University. The animals were grouped (six rats/cage), under standard laboratory conditions with water and food provided ad libitum. All animal experiments were performed following the ethical standards according to the *Guide for the Care and Use of Laboratory Animals* of the National Institutes of Health (Institute of Laboratory Animal Resources 1996) in the Faculty of Medicine, Alexandria University, Egypt.

**Figure 1:**
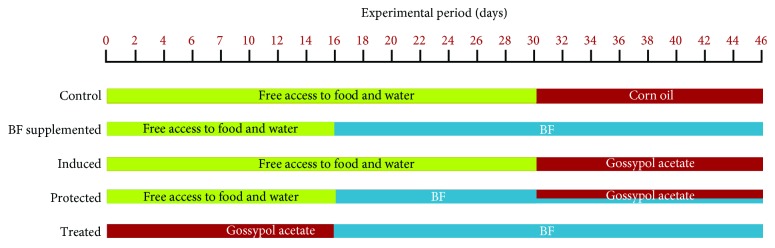
An illustration of the experimental design groups. Corn oil (0.5 ml, intraperitoneally) eight times for 16 days, BF (100 mg/kg BW, orally by gavage) daily for 30 days, and gossypol acetate (5 mg/kg BW, intraperitoneally, dissolved in corn oil) eight times for 16 days. Green colour indicates free access to food and water, red colour indicates the administration of corn oil or gossypol acetate, and blue colour indicates treatment with BF.

The healthy experimental animals were equally divided into five groups (Figure 1). Group 1 (control) received corn oil (0.5 ml, intraperitoneally) eight times for 16 days. Group 2 (BF supplemented) received BF (100 mg/kg BW, orally by gavage) daily for 30 days. Group 3 (induced) received gossypol acetate (5 mg/kg BW, intraperitoneally, dissolved in corn oil) eight times for 16 days. Group 4 (protected) was administered BF alone for 2 weeks and then was coadministered with gossypol acetate for the next 16 days. Group 5 (treated) received gossypol acetate for 16 days and then was treated with BF for 30 days. The doses of BF and gossypol acetate were as mentioned in groups 2 and 3, respectively (Figure 1). Experimental animals in groups 1, 2, 3, and 4 were allowed free access to water and food without any treatments until the 16th day for groups 2 and 4 for and until the 30th day for groups 1 and 3.

At the end of the experiment (after 46 days), the rats were fasted for eight hours and then the blood was collected from the eye canthus to measure the blood glucose level. Rats were allowed to complete fasting overnight and then decapitated to collect the blood and testes. Sera were isolated and stored at −20°C.

### 2.4. Preparation of Testicular and Epididymal Tissues

After decapitation, one testis was removed and most of the parenchyma (2/3) was weighed. A 10% (w/v) homogenate of testis tissues in 0.1 M phosphate buffer saline (PBS), pH 7.4, was prepared by using a mortar in an ice bath and centrifuged at 10,000*g* for 20 min at 4°C. The supernatant was collected and stored for further biochemical investigations. The second testis and one epididymis were postfixed overnight in 10% neutral buffered formalin for histopathological study. The second epididymis was isolated, washed, crushed in 2 ml Ham's F-10 medium (0.5% bovine serum albumin, BSA), and incubated at 37°C for the estimation of the spermatozoal quality by automated examination by MiraLab's Computer Aided Semen Analysis System (CASA, WLJY 9000, Beijing Weili New Century Science and Tech. Dev. Co. Ltd., China) [39]. Methylene blue and eosin red stains were used, respectively, for studying the sperm morphology.

### 2.5. Biochemical Assays

Serum-reduced glutathione (GSH) and TBARS as well as testicular TBARS and nitric oxide levels were determined according to Jollow et al. [40], Tappel and Zalkin [41], and Menaka et al. [42], respectively. Testosterone level was determined in serum by using an ELISA commercial kit [43], and the levels of glucose [44], cholesterol [45], and albumin [46] were also measured in the serum by using commercial kits (Biosystems S.A., Spain).

### 2.6. Semen Parameters

Sperm count, motility, and morphology index were assessed using CASA. Alpha-glucosidase activity and fructose level are important parameters related to semen quality. Alpha-glucosidase activity was measured by using the method of Han and Srinivasan [47], in which the specific activity (IU/mg) of the enzyme was defined as micromoles (*μ*mol) of *p*-nitrophenol released per min per milligram (mg) of protein. The semen fructose level was determined according to Foreman et al. [48].

### 2.7. Determination of Sperm Inflammatory Markers

Testicular IL-1*β*, IL-6, IL-12, and IL-18 were estimated by using enzyme-linked immunosorbent assay (ELISA) kits (Koma Biotech–Korea), and testicular TNF-*α*, ADAM-17, and TIMP-3 were determined by Sun Red (England) ELISA kits. Precoated wells with the captured antibodies were washed four times with the washing buffer. Standard or samples (100 *μ*l) were added to each well in duplicate, covered, and incubated at RT for 2 h. The plates were then washed four times and 100 *μ*l of the diluted detection antibody was added per well, covered, and incubated at RT for 2 h. 100 *μ*l of streptavidin-HRP was added to each well and incubated for 30 min at RT for a proper colour development. The plates were washed and 100 *μ*l of the substrate solution (3,3′,5,5′-tetramethylbenzidine, TMB) was added to each well and incubated for 30 min at RT. The reaction was terminated by adding 100 *μ*l of the stop solution (H_2_SO_4_, 5%) to each well, and the colour developed was read at 450 nm on a plate reader (Sanofi Diagnostics Pasteur, France).

### 2.8. Histopathological Changes

The testes and epididymides of the control and experimental groups were removed, postfixed overnight in 10% neutral buffered formalin, dehydrated in ascending grades of alcohol (70%, 80%, 95%, and absolute alcohol), and cleaned by immersion in xylene followed by impregnation in melted paraffin wax for 1–2 h. Sections 5 *μ*m thick were cut by using a rotary microtome. Finally, the sections were stained with conventional hematoxylin and eosin (H&E) stain for examination under a light microscope of any histopathological changes. The histopathological study was carried out in the Histopathology Department, Faculty of Medicine, Alexandria University.

### 2.9. Statistical Analysis

Data was analysed by one-way analysis of variance (ANOVA) using the Primer of Biostatistics (Version 5) software program. The significance of means ± SD was detected between groups by a post hoc test (Tukey) at *p* < 0.05.

## 3. Results

### 3.1. Characterization of Berberine-Rich Fraction

**Figure 2:**
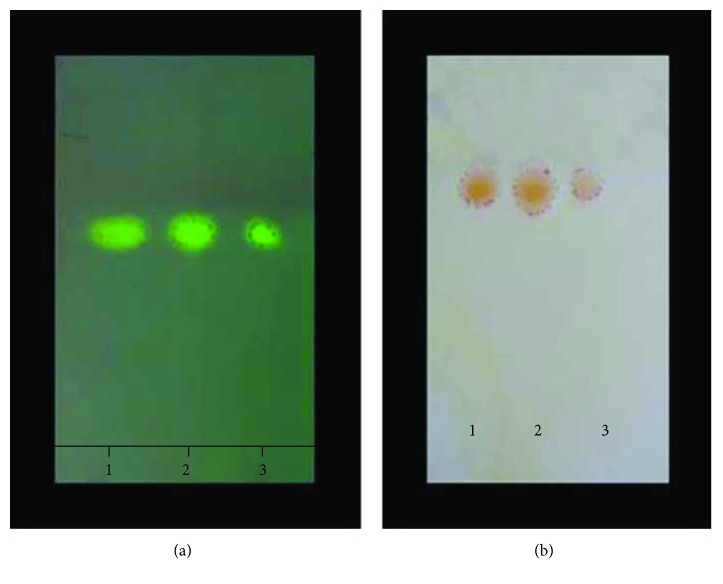
TLC identification of standard berberine chloride (spot 1), chloroform fraction (spot 2), and isolated berberine base (Spot 3).

Table 1 shows the berberine concentration in different prepared samples, and BF had the highest concentration (0.89 mg/mg extract). The melting point of berberine chloride was 190°C and that of the berberine base was 165°C. Both the chloroform fraction and BF had the same berberine spot equal distance as shown in TLC results in Figure 2.  Table 2 presents the ^1^H-NMR (DMSO/TMS) showing *δ*: 3.17 (2H, t, H 5), 4.03 (3H, s, H 10-OCH_3_), 4.05 (3H, s, H 9-OCH_3_), 4.89 (2H, t, *J* = 5.35 Hz, H 6), 6.14 (2H, s, 3-OCH_2_O), 7.06 (1H, s, H 4), 7.77 (1H, s, H 1), 7.96 (1H, d, H 12), 8.17 (1H, d, *J* = 8.4 Hz, H 11), 8.90 (1H, s, H 13), and 9.85 (1H, s, H 8) as our team previously published [36].

### 3.2. Effect of Berberine-Rich Fraction on Blood Parameters

Serum GSH, TBARS, testosterone, cholesterol, glucose, and albumin levels were measured to assess the effect of BF alone or in combination with gossypol on rats' fertility. The administration of BF to healthy rats showed no significant change in the levels of GSH, testosterone, or albumin compared to the normal control group. While it significantly decreased the levels of TBARS, cholesterol, and glucose compared with those of the control group as shown in Table 3, gossypol acetate-injected rats (induced group) had a significantly increased TBARS level and decreased GSH, testosterone, cholesterol, glucose, and albumin levels compared to the control group. BF coadministration with gossypol acetate (protected group) significantly decreased the TBARS level and increased GSH, testosterone, cholesterol, glucose, and albumin levels compared to the induced group, at *p* < 0.05. The treatment with BF (treated group) normalized the TBARS level and significantly enhanced the GSH, testosterone, cholesterol, glucose, and albumin levels, approaching the control levels (Table 3).

### 3.3. Effect of Berberine-Rich Fraction on Semen Quality

**Figure 3:**
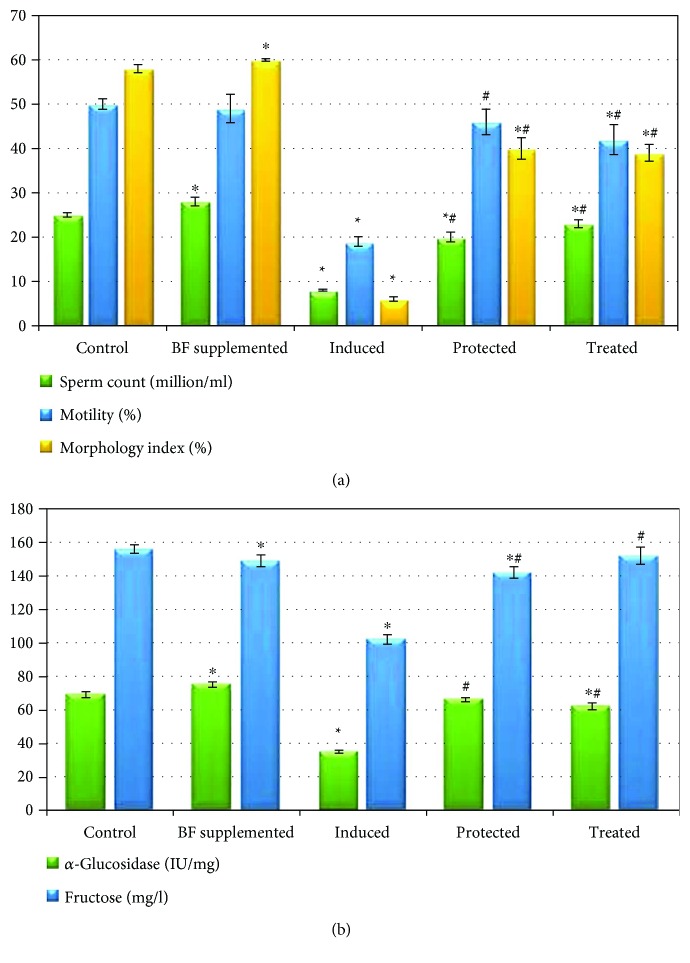
Effects of berberine-rich fraction on sperm parameters. Graph (a) shows sperm count, motility, and morphology index. Graph (b) shows *α*-glucosidase activity and fructose level. Values represent the mean ± SD of 6 rats. ^∗^*p* ≤ 0.05 versus control group. ^#^*p* ≤ 0.05 versus induced group. One-way ANOVA followed by Tukey's test was used.

The BF-supplemented group showed normal sperm motility and had significantly improved sperm count and morphology compared with the control group. In addition, BF administration significantly increased the *α*-glucosidase activity compared to that of the control group. The male infertility-induced group showed a highly significant decrease in the sperm count, inhibited sperm motility, and a marked change in the sperm morphology to irregular shapes compared to the control group. Gossypol acetate injection also significantly inhibited the *α*-glucosidase activity and significantly decreased the semen fructose level compared with the control group. The BF-protected and BF-treated groups had significantly improved sperm count, motility, and morphology as well as significantly increased *α*-glucosidase activity and semen fructose level compared to the induced group (*p* < 0.05), with their values approaching the values of the control group, as shown in Figure 3.

### 3.4. Effect of Berberine-Rich Fraction on the Testicular Inflammatory Markers

**Figure 4:**
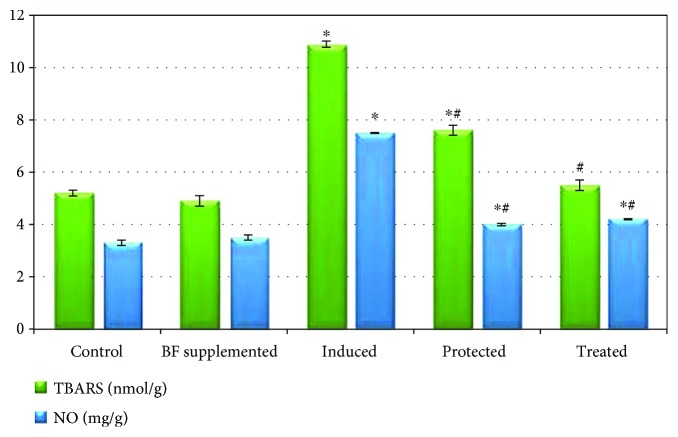
Effect of berberine-rich fraction on sperm prooxidants. Values represent the mean ± SD of 6 rats. ^∗^*p* ≤ 0.05 versus control group. ^#^*p* ≤ 0.05 versus induced group. One-way ANOVA followed by Tukey's test was used.

**Figure 5:**
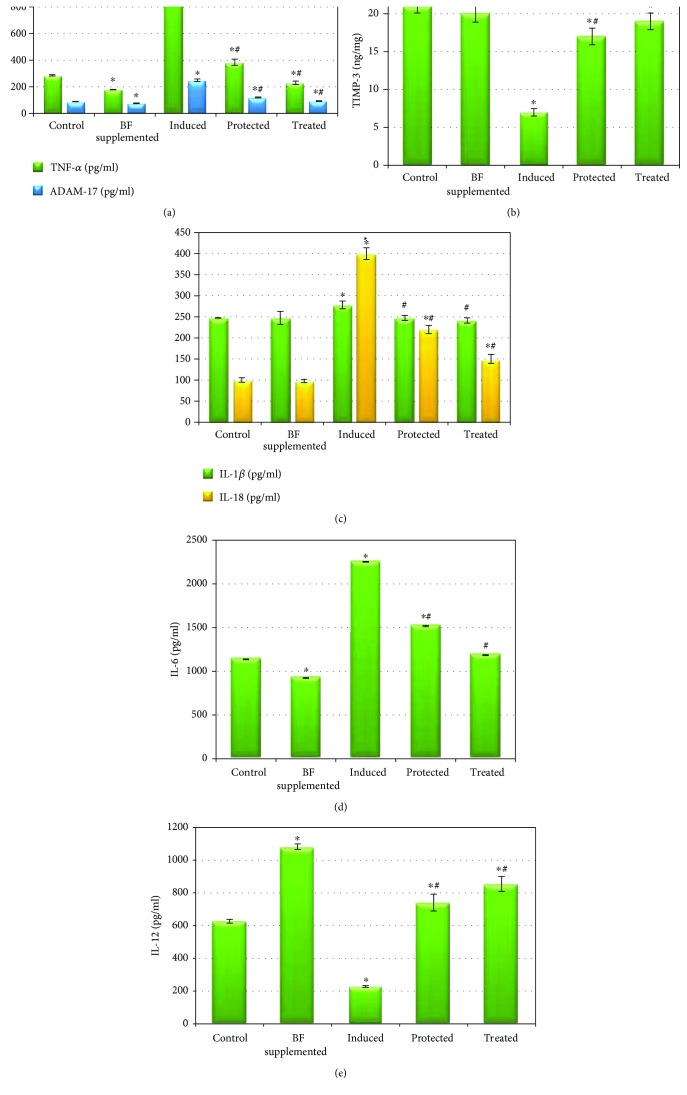
Effect of berberine-rich fraction on sperm inflammatory markers. Values represent the mean ± SD of 6 rats. ^∗^*p* ≤ 0.05 versus control group. ^#^*p* ≤ 0.05 versus induced group. One-way ANOVA followed by Tukey's test was used.

Figures 4 and 5 demonstrate that the administration of BF to healthy rats resulted in a nonsignificant change in the levels of testicular TBARS, NO, TIMP-3, and interleukins (IL-1*β* and IL-18) compared to the normal control group, at *p* < 0.05. It also significantly decreased TNF-*α*, ADAM-17, and IL-6 while it increased IL-12 compared to that of the control group (at *p* < 0.05). Gossypol-induced male infertility markedly and significantly increased the levels of testicular TBARS, NO, TNF-*α*, ADAM-17, and interleukins (IL-1*β*, IL-6, and IL-18) while significantly decreasing the levels of both TIMP-3 and IL-12 compared with those of the control group. A significant decrease in the levels of testicular TBARS, NO, ADAM-17, TNF-*α*, and interleukins (IL-18, IL-6, and IL-1*β*) as well as a significant increase in the levels of TIMP-3 and IL-12 was observed in the BF-protected and BF-treated groups compared to the gossypol-induced group. In addition, the administration of BF after gossypol injection (treated group) normalized the levels of testicular TIMP-3, TBARS, and interleukins (IL-1*β* and IL-6), approaching the values of the control group.

### 3.5. Histological Changes in Testicular and Epididymal Tissues of Different Experimental Groups

**Figure 6:**
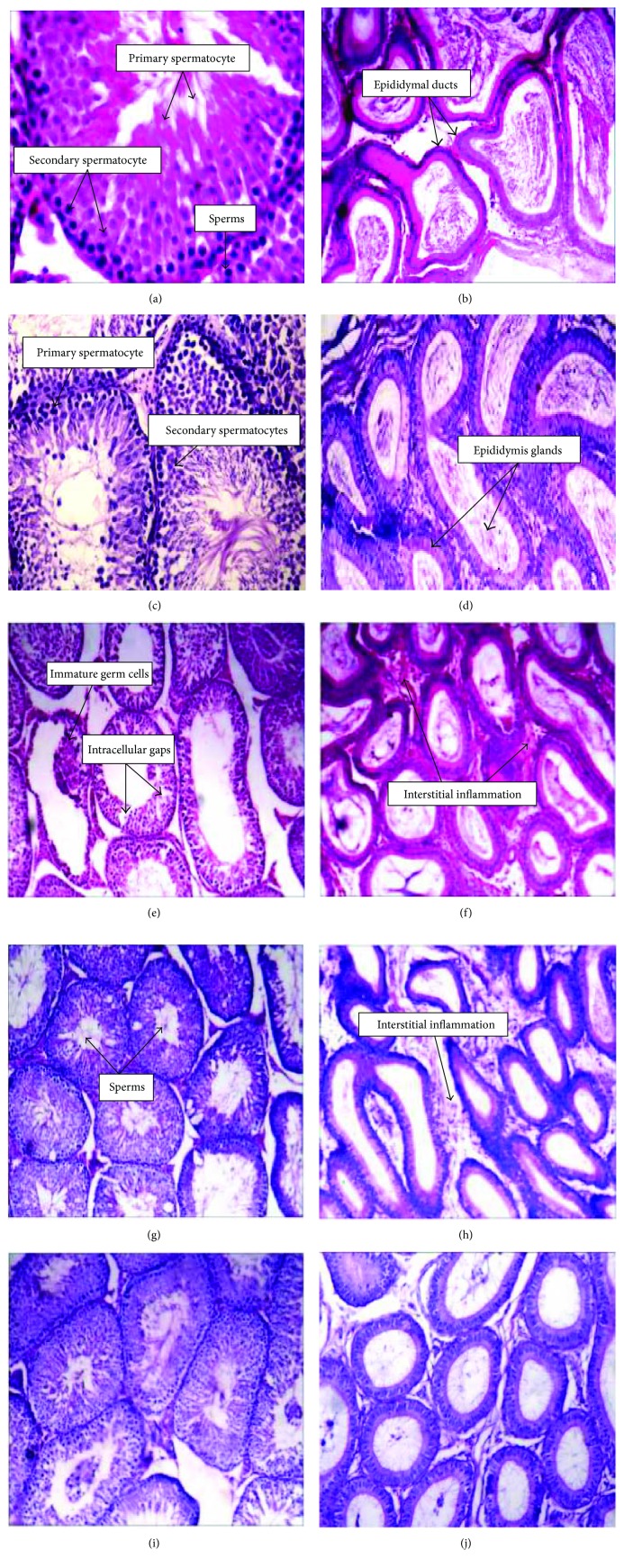
The histological examination of both testicular and epididymal tissues of different treated groups compared to the control one. (Magnification of a and c: ×40; magnification of b, d, e, f, g, h, i, and j: ×10). Testicular (a) and epididymal tissues (b) of control group. Testicular (c) and epididymal tissues (d) of berberine-rich fraction (100 mg/kg orally) supplemented group. Testicular (e) and epididymal tissues (f) of gossypol-induced group (5 mg/kg, 8 times). Testicular (g) and epididymal tissues (h) of berberine-rich fraction (100 mg/kg orally) protected group. Testicular (i) and epididymal tissues (j) of berberine-rich fraction (100 mg/kg orally) treated group.

Figures 6(a)–6(d) show that the control healthy and BF-supplemented rats had normal and well-organized seminiferous tubules, and they show all stages of spermatogenesis till the stage of sperm formation. They also have normal epididymal ducts. On the other hand, the testicular tissue of the gossypol-treated group (Figure 6(e)) shows an accumulation of immature germ cells in the lumen and defects in spermatogenesis and sperm formation. Furthermore, it shows an increase in intracellular gaps due to disruption in cell-cell contacts in the seminiferous epithelium compared to the control one. Moreover, the epididymal section (Figure 6(f)) revealed leukocytic infiltration, congestion, and edema. The BF-protected group shows almost near-normal spermatogenesis with sperm formation in the testicular sections (Figure 6(g)) and mild interstitial inflammation and edema in the epididymal sections (Figure 6(h)). The testicular section of the BF-treated group (Figure 6(i)) revealed a marked improvement in spermatogenesis in all stages till sperm formation compared with those observed in the control one, but little interstitial inflammation was observed in the epididymal section (Figure 6(j).

## 4. Discussion

Male infertility is considered to be one of the most critical health problems that are expected to increase [49]. Multiple lifestyle and environmental factors contributed to the etiology of male infertility including cigarette smoking, alcohol, heavy metals, pesticides, radiation, and illicit drugs; these factors are growing in number and are widely spread [50, 51]. In this study, BF (89%) was shown to be a leading therapy for this problem by controlling the inflammatory markers that are produced in the case of male infertility and that result in a destructive damage to sperm and reduction in the semen quality.

Gossypol is a toxic phenolic compound extracted from cottonseeds [28, 38]. Deleterious effects of gossypol on fertility have been widely reported in the literature [29, 52]. The intraperitoneal injection of gossypol acetate significantly reduced the semen quality as evidenced by the decrease in the sperm count and motility and changed the sperm morphology as well as inhibited the *α*-glucosidase activity compared with those of the control group. Alpha-glucosidase activity is widely used as an indicative marker for sperm count as alpha-glucosidase is produced by the epididymis, so a low level of *α*-glucosidase indicates epididymal obstruction [53]. Gossypol administration significantly reduced the enzyme activity in all segments of the epididymis [54]. The major antifertility effect of gossypol is to inhibit sperm production and motility; this inhibitory action has been attributed to a dramatic drop in the production of mitochondrial ATP [29]. Furthermore, gossypol induces oxidative stress by promoting the formation of ROS and lipid peroxides, which negatively affected plasma membrane permeability, ATPase activity, and glucose transport in sperms [55, 56]. In the present study, gossypol injection significantly elevated serum and testicular TBARS and NO levels as well as depleted the antioxidant capacity (GSH) and serum glucose level, resulting in the damage of sperm membrane. These results are in concert with the reports from El-Sharaky et al. [38] and Santana et al. [29] that demonstrated the reproductive damage caused by gossypol-induced oxidative stress in rats and that from Chen et al. [52] that reported a lowered serum glucose level after gossypol treatment.

Free radical overproduction enhances proinflammatory gene expression and is associated with inflammatory reactions. On the other hand, inflammatory cells increased the generation of ROS, leading to exaggerated oxidative stress that impairs sperm function [8, 11]. The proinflammatory cytokines TNF-*α*, IL-1*α* and IL-1*β* may have certain physiological functions in the male genital tract. However, the elevated levels of these cytokines compared with the normal one, as seen during the inflammation process, are very harmful to sperm production [8]. Increasing the generation of ROS induces ADAM-17 expression and results in TNF-*α* proteolytic cleavage, which in turn increases ROS production, induces oxidative stress, promotes lipid peroxidation, causes destructive damage sperm cell membrane, decreases sperm motility, and induces apoptosis [57]. TIMP-3 is an inhibitor for ADAM-17 and negatively controls its activity [15]. Thus, the balance between ADAM-17 and TIMP-3 expression can control the inflammation process. In addition, IL-18 was found to stimulate the *cytotoxic* activity of T cells and natural killer cells and stimulates the production of IL-6 and TNF-*α* [58]. On the other hand, Naz and Evans [12] suggested a significant correlation between IL-12 levels and fertility as these improve the count and normal morphology of sperm in the semen. Therefore, male infertility may be attributed to its derangement. The destruction of testicular tissues, in the case of infertility, lowered the production of IL-12 and negatively affected the sperm count and motility [12]. In agreement with the earlier findings, gossypol acetate injection significantly increased the levels of testicular ADAM-17, TNF-*α*, and interleukins (IL-1*β*, IL-6, and IL-18), while it decreased TIMP-3 and IL-12 levels compared with those of the control group. TNF-*α* affects the androgenic receptor controlling testosterone activity; it decreases the production of testosterone and reduces the sperm function [2].

Testosterone, as a steroid hormone, is essential for spermatogenesis [5]. Gossypol was found to induce the regression of Leydig cells, resulting in a significant decrease in the level of serum testosterone and reduced libido [56]. Furthermore, testosterone regulates the formation of seminal fructose, as it controls the activity and function of the accessory glands that are responsible for fructose secretion. Fructose is an important source of energy for sperms and is required for sperm motility [59]. Thus, the decreased level of semen fructose is due to gossypol-induced depletion in the testosterone level. In addition, gossypol decreased the serum level of cholesterol, which is consistent with the studies by Nwoha and Aire [60] and Obeidy et al. [61], who reported that gossypol altered the serum lipoprotein metabolism.

Antioxidants can contribute to the protection of cells and tissues against the deleterious effects of free radicals [9]. The toxicological effect of gossypol was reversed by the treatment of animals with berberine-rich fraction. Berberine (BBR) is one of the most potent ingredients in *Berberis vulgaris*, characterized by a diversity of pharmacological effects [17, 22] including antioxidant and anti-inflammatory properties in a variety of tissues including kidney, liver, pancreas, and adipose tissue. BBR administration reduced the oxidative stress markers (TBARS) and evaluated the antioxidant enzymes (GSH, GPx, and SOD) in diabetic animals [24]. In the present study, the oral administration of BF successfully reversed most of the hazardous effects of gossypol on semen characteristics. BF significantly enhanced the sperm count, motility, and morphology as well as improved *α*-glucosidase activity and increased the semen fructose level. BF has antioxidant properties, as confirmed by the reduction of TBARS and NO levels as well as the elevation of the reduced level of glutathione, and can be considered one of the most potent antioxidant agents, protecting the cell against ROS destructive damage [62]. These results confirmed the potent antioxidant capacity of BF as mentioned previously [27, 63, 64].

The oral administration of BF either with or after gossypol acetate injection represented an ameliorative effect against the analysed inflammatory markers. Previously, BBR showed a potent anti-inflammatory effect by suppressing the production of inflammatory mediators such as TNF-*α*, COX-2, IL-1*β*, IL-6, NO, and inducible nitric oxide synthase (iNOS) as well as by the inhibition of arachidonic acid metabolism [23, 24, 65, 66]. In addition, BBR was found to inhibit the activator protein-1 (AP-1, a key transcription factor in inflammation) [67] and inhibit DNA synthesis in active lymphocytes, resulting in the inhibition of lymphocyte transformation [68].

The obtained biochemical results were confirmed by the histological study where gossypol intraperitoneal injection resulted in a marked histological alterations in the testicular and epididymal tissues including depressed spermatogenesis, degenerative germ cells, and vacuoles (in testicular section) as well as leukocytic infiltration and edema (in epididymal section), which are in agreement with the El-Sharaky et al. [38]. A normal histological structure of a rat's testis and epididymis was observed in both control and BF-supplemented animals. Almost near-normal spermatogenesis with sperm formation in the testicular sections and little mild interstitial inflammation in the epididymal sections were observed in BF-protected and BF-treated groups.

In conclusion, the mechanism of gossypol-induced toxicity on rats' testes contributed to the induction of oxidative stress and inflammatory responses, leading to cell membrane damage and reduced sperm count, motility, and morphology. The administration of BF to animals was shown to be effective in preventing oxidative damage and inflammation induced by gossypol. Thus, the use of BF can be suggested as a palliative measure in animals subjected to poisoning by gossypol. However, further studies must be done to confirm the anti-inflammatory effect of BF by using different experimental models. Moreover, the concentration of active metabolite (berberine) must be measured in the testicular tissue and the pharmacokinetics of BF should be investigated in rats.

## Figures and Tables

**Table 1:** The berberine concentration in different prepared samples.

Extract	Berberine concentration (mg/mg extract)
Ethanolic extract	0.6
Chloroform fraction	0.73
Berberine-rich fraction	0.89

**Table 2:** ^1^H-NMR (DMSO/TMS) chemical shifts of isolated berberine in ppm [36].

Proton	*δ* (ppm)
H 5	3.17 (2H, t)
H 10-OCH_3_	4.03 (3H, s)
H 9-OCH_3_	4.05 (3H, s)
H 6	4.89 (2H, t, *J* = 5.35 Hz)
H 2,3-OCH_2_O	6.14 (2H, s)
H 4	7.06 (1H, s)
H 1	7.77 (1H, s)
H 12	7.96 (1H, d, *J* = 8.4 Hz)
H 11	8.17 (1H, d, *J* = 8.4 Hz)
H 13	8.90 (1H, s)
H 8	9.85 (1H, s)

**Table 3:** Effect of berberine-rich fraction on blood parameters of different experimental groups.

Groups	TBARS (nmol/ml)	GSH (mg/ml)	Testosterone (ng/ml)	Cholesterol (mg/dl)	Glucose (mg/dl)	Albumin (g/dl)
Control	0.49 ± 0.01	0.38 ± 0.01	3.7 ± 0.21	110 ± 5.2	95 ± 2.4	3.5 ± 0.2
BF supplemented	0.44 ± 0.02^∗^	0.37 ± 0.02	3.8 ± 0.15	90 ± 1.6^∗^	80 ± 3.1^∗^	3.4 ± 0.1
Induced	0.58 ± 0.03^∗^	0.29 ± 0.01^∗^	0.36 ± 0.23^∗^	65 ± 2.3^∗^	65 ± 2.1^∗^	2.9 ± 0.04^∗^
Protected	0.52 ± 0.03^#^	0.32 ± 0.06^#^	3.2 ± 0.34^#^	75 ± 1.9^∗#^	75 ± 2.8^∗#^	3.3 ± 0.05 ^#^
Treated	0.49 ± 0.05^#^	0.30 ± 0.08^#^	3.4 ± 0.16^#^	85 ± 3.9^∗#^	82 ± 2.6^∗#^	3.1 ± 0.2^#^

Values represent the mean ± SD of 6 rats. ^∗^*p* ≤ 0.05 versus control. ^#^*p* ≤ 0.05 versus induced group. ANOVA (one-way) followed by Tukey's test.
